# Minimally Invasive Surgery for Early-Stage Nasopharyngeal Carcinoma

**DOI:** 10.1097/SCS.0000000000008765

**Published:** 2022-07-26

**Authors:** Jinping Liu, Zesheng Zeng, Dingting Wang, Gang Qin

**Affiliations:** Department of Otolaryngology Head and Neck Surgery, The Affiliated Hospital of Southwest Medical University, Luzhou, China

**Keywords:** Endoscopic nasopharyngectomy, endoscopic surgery, minimally invasive surgery, nasopharyngeal carcinoma, robot-assisted surgery

## Abstract

According to the National Comprehensive Cancer Network guidelines, the preferred treatment for early-stage nasopharyngeal carcinoma (NPC) is radiotherapy, however, the toxic effects associated with radiotherapy have been a nuisance for patients. Minimally invasive surgery for recurrent NPC has been widely recognized as an effective way to completely remove the tumor and free the patient from or mitigate the toxicity of radiotherapy. Therefore, some researchers hope that minimally invasive surgery can be used to treat early-stage NPC. It is a bold and controversial attempt, and the researchers’ efforts have achieved initial results. This article reviews the preliminary results of minimally invasive surgery for NPC, especially the feasibility and challenges of minimally invasive surgery for early-stage NPC.

Nasopharyngeal carcinoma (NPC) is an epithelial malignancy originating from the mucosal epithelium of the nasopharynx. According to the World Health Organization, NPC is divided into 3 pathological subtypes: squamous cell carcinoma, nonkeratinized carcinoma, and basal-like squamous cell carcinoma. Because of its sensitivity to radiotherapy, intensity-modulated radiation therapy (IMRT) has become the preferred treatment of NPC. However, the acute and chronic toxic reactions caused by radiotherapy, such as mucositis, dry mouth, tooth loss, acquired posterior nostril stenosis, radioactive encephalopathy, and radioactive osteonecrosis, have seriously reduced the quality of life of patients and even affected the survival rate of patients.^1^,^2^ In a 15-year follow-up study,^2^ almost all patients with early-stage NPC who received IMRT suffered from grades 1 to 3 acute toxicity, including mucositis (100%), radiodermatitis (99.5%), and xerostomia (96.3%). Subcutaneous fibrosis (89.8%) and deafness or otitis (72.2%) were the most common late toxicities. In addition, even after reducing the radiation therapy clinical target volume and dose, 54.4% of patients still suffered grade 2 or more radiotherapy toxicity.^3^

As a minimally invasive surgical technique, endoscopic surgery can make use of natural or artificial tract such as nasal cavity, throat, digestive tract, and thoracic cavity to perform delicate surgical operations, and remove lesions without external incision or with only a small incision.^4^–^6^ It not only can avoid the huge trauma caused by open surgery, reduces the pain of the patients, and accelerates the recovery of the patients after surgery,^6^ but also can avoid or alleviate patients suffering from radiation therapy toxicity.^7^ The nasopharynx is located behind the nasal cavity and can be reached through a nasal endoscopy. However, NPC usually occurs in the center of the skull base region and is adjacent to important nerves and blood vessels such as carotid artery, brain stem, and optic nerve. The difficulty of operation restricts the development of endoscopic application in the surgical treatment of NPC. In recent years, with the in-depth understanding of anatomy and the continuous improvement of surgical skills, endoscopic surgery has been gradually applied in the surgical treatment of NPC as an auxiliary or main surgical method, which can avoid or alleviate patients suffering from radiation therapy toxicity while completely removing tumor.

## REVIEW OF ENDOSCOPIC SURGERY IN LOCALLY RECURRENT NASOPHARYNGEAL CARCINOMA

According to the published literature, 10.0% to 36.0% of patients with NPC recur after initial treatment,^8^ and the National Comprehensive Cancer Network guidelines recommend that salvage surgery should be the treatment of choice for all locally recurrent nasopharyngeal carcinoma (rNPC), when possible. A recent meta-analysis included 22 studies of 1186 patients with rNPC or residual NPC,^9^ showed that the patients with locally rNPC or residual NPC who underwent endoscopic surgery had significantly improved survival compared with open surgery. In addition, patients who accepted open surgery were also more prone to serious complications, such as trismus, palatal fistula, cleft palate, nerve injury, osteonecrosis, flap necrosis, cerebrospinal fluid rhinorrhea, dysphagia, etc. The 2 most common complications were trismus and palatal fistula, accounted for 40.3% and 36.4% of all complications. The top 2 complications in patients underwent endoscopic surgery were otitis media and osteonecrosis, accounted for 45.0% and 11.2% of all complications, and fatal complications were rare.

In a multicenter randomized-controlled clinical trial comparing the efficacy and safety of endoscopic salvage surgery and IMRT in patients with locally rNPC,^7^ 200 patients were randomly divided into endoscopic nasopharyngectomy (ENPG) group or IMRT group (1:1). A total of 96 of 100 patients in the ENPG group received ENPG, and all patients in the IMRT group received IMRT. The results showed that during the follow-up period of 42 to 69 months, a total of 74 patients died, of which 29 in the ENPG group were lower than 45 in the IMRT group. The 3-year overall survival rate in the ENPG group was 85.8%, which was significantly higher than that in the IMRT group (68.0%), and the 3-year disease-free survival and local recurrence survival were also better. This suggests that endoscopic surgery significantly improves overall survival compared with IMRT in patients with resectable locally rNPC.

The successful application of endoscopic surgery in locally rNPC has also led surgeons to further consider whether it is possible to apply these successful experiences in the treatment of early-stage NPC, which is a bold and innovative idea, and some researchers have put this idea into practice.

## APPLICATION OF ENDOSCOPIC SURGERY IN EARLY-STAGE NPC

There are few publications on endoscopic surgery in the treatment of early-stage NPC (Supplemental Table 1, Supplemental Digital Content 1, http://links.lww.com/SCS/E220). The scholars have used single endoscopic surgery or endoscopic surgery combined with chemotherapy or chemoradiotherapy to explore the efficacy and safety of endoscopic surgery in the treatment of early-stage NPC.

Huang and Qiu^10^ applied endoscopic surgery for the first time in the treatment of 10 patients with early-stage NPC. All patients received 4 courses of cisplatin, paclitaxel and 5-fluorouracil chemotherapy after endoscopic surgery and were followed up for 9 to 128 months, with a 2-year survival rate of 100%, local recurrence occurred in 1 patient, and no postoperative complications such as dry mouth and sticky saliva occurred.

Chen et al^11^ introduced a novel ENPG and described it in details,^12^ including: defined the tumor invasion regions and surgical margins for high-risk microinvasion regions. The area of tumor invasion plus an additional 0.5 to 1.0 cm of peripheral mucosal margin and 2 to 3 mm of basal margin of the sphenoid bone surface and cranial base ramp were defined as the surgical margins, which the surgeons need to strictly follow to remove the tumor during dissection. The team successfully used the technology to treat rNPC.^13^,^14^ Based on the above foundation, Liu et al^15^ collected 339 patients with stage I NPC from 2007 to 2017, and performed ENPG alone for 10 patients who refused radiotherapy, the remaining 329 patients received IMRT. All patients undergoing surgery must meet the following conditions: first, the primary tumor or tumor base is <1.5 cm. Second, the tumor is at least 0.5 cm away from the internal carotid artery. Third, the retropharyngeal lymph node is <0.4 cm, and the cervical lymph node is <0.6 cm. The results showed that after a median follow-up of 59 months, none of the 10 patients who received ENPG had died or had local recurrence or metastasis, with a 5-year overall survival rate of 100%, local recurrence-free survival rate of 100%, regional recurrence-free survival rate of 100%, and the distant metastasis-free survival rate of 100%, which were similar to the IMRT group (99.1%, 97.7%, 99.0%, 97.4%). However, compared with IMRT group, the ENPG group had less cost of hospitalization and improved quality of life scores such as pain, swallowing, dry mouth, and thicky saliva.

Zhang et al^16^ explored the feasibility and safety of ENPG combined with low-dose radiotherapy (LDRT) in the treatment of early NPC by referring to the above surgical methods.^12^,^15^ A total of 37 patients with localized T1-2 NPC in the experimental group were treated with ENPG+LDRT program (ENPG+LDRT group), meanwhile, they recruited 132 T1-2 NPC patients in the control group treated with IMRT (IMRT group). With a mean follow-up time of 54 months, only 1 patient in the ENPG+LDRT group died of hepatic metastases. There were no significant differences in 5-year overall survival, distant metastasis-free survival, local recurrence-free survival, and regional recurrence-free survival in the ENPG group compared with IMRT group (97.3% versus 97.7%, 97.3% versus 90.2%, 100% versus 95.5%, 100% versus 97.0%, all *P*>0.05), but patients’ quality of life and the incidence of late radiotherapy-related sequelae were lower.

Weng et al^17^ reported the short-term and long-term prognostic effects of ENPG combined with chemoradiotherapy of early-stage NPC. Fifty-eight patients with ENPG combined with chemoradiotherapy (surgery group) and 98 patients with conventional chemoradiotherapy (nonsurgery group) were matched by a propensity score of 1:2. The results showed that the 5-year overall survival rate, disease-free survival rate, and relapse-free survival rate in the surgery group were higher than those in the nonsurgery group (98.3% versus 91.7%, 98.3% versus 81.4%, 100% versus 90.1%). At the end of therapy, there were no cancer residues in the surgical group compared with 14 in the nonsurgical group, and the surgery group also had a lower incidence of severe oral mucositis.

## A NEWLY MINIMALLY INVASIVE SURGERY: ROBOT-ASSISTED SURGERY

With the rapid development of artificial intelligence, robot-assisted surgery (RAS) has also been gradually applied in otolaryngology head and neck surgery.^18^–^20^ Ozer and Waltonen^21^ first described a robotic-assisted transoral soft palate incision for NPC resection in a cadaveric model to achieve full exposure of the nasopharynx and localize and dissect the internal carotid artery without making an external incision, then stitched with the help of a robot. Tsang et al^22^ performed nasopharyngectomy for 12 patients with rNPC using the da Vinci surgical robot. The results showed that the operative time was comparable to that of open surgery, with a median operative time of 225 minutes. The survival rate was 83.0%, and the disease-free survival rate was 61.0%. Ding et al^19^ applied transoral robotic surgery (TORS) retropharyngeal lymph node dissection in NPC patients with retropharyngeal lymph node recurrence, negative margins were obtained in all patients, en bloc resection was achieved in 8 of 10, and only 1 patient experienced recurrence at 19-month follow-up. RAS makes minimally invasive surgery for head and neck cancers safer and more feasible by providing intraoperative flexible robotic arms and 3-dimensional views. We conjecture that RAS may also be used in the treatment of early-stage NPC in the future.

## CHALLENGES AND PROSPECTS OF MINIMALLY INVASIVE SURGERY FOR EARLY-STAGE NPC

Although endoscopic surgery for rNPC has been accepted by the public and has achieved good results, there are still few studies on endoscopic surgery for early-stage NPC,^10^,^15^–^17^ may due to the more practical clinical problems: first, radiotherapy is still the first-line therapy for early-stage NPC, and its high cure rate cannot be ignored; second, the anatomical position of the nasopharynx is hidden and adjacent to important blood vessels and nerves, so the anatomical theory and surgical skills of the operator are highly required. Third, in the existing reports, the sample size of endoscopic surgery for early-stage NPC is small, and there is no convincing big data or multicenter clinical trials to confirm its reliability. In addition, some researchers^23^ pointed out that most of the cases reported in the current literature were carefully selected, and the screening process was very complicated, especially for the specific requirements of lymph node size, which might not be universally applicable.

Although some researchers are skeptical or even negative about the efficacy of endoscopic surgery in the treatment of early-stage NPC, it does not mean that this treatment option is completely infeasible. First of all, with the improvement of people’s health awareness and the application of screening technologies such as Epstein-Barr Virus liquid biopsy,^24^ magnetic resonance imaging,^25^ and artificial intelligence screening,^26^ about 41.7% of early NPC limited to nasopharyngeal cavity have been found,^27^ which provides the possibility of complete resection of tumors by endoscopic surgery. Second, the implementation of endoscopic technology training and indepth understanding of anatomy have improved the surgical skills of surgeons. Endoscopic surgery is no longer only used to treat inflammatory diseases of the nasal cavity and sinus, but also gradually applied to the complex nasopharyngeal-skull base anatomy.^28^ In addition, artificial intelligence-assisted technology^26^,^29^ and 3-dimensional navigation technology^30^,^31^ also guide and help surgeons to perform delicate and accurate dissection. All these factors provide favorable conditions for endoscopic surgery in the treatment of early-stage NPC. However, it is worth noting that the application of minimally invasive surgery to early-stage NPC should be very cautious. Strict selection of cases, superb skills of the operators, ensuring the most negative margins, and accurate postoperative follow-up treatment are all necessary indeed.

## CONCLUSION

In summary, the current study indicates that minimally invasive surgery is feasible for the treatment of early-stage NPC, and its short-term efficacy is encouraging, but its long-term efficacy needs further observation, and multicenter and large-sample clinical studies are needed to further verify the reliability of the efficacy. Minimally invasive surgery provides a new option for patients with early-stage NPC, especially those who refuse radiotherapy, and it may become a new direction for the treatment of early-stage NPC in the future.

## Supplementary Material

SUPPLEMENTARY MATERIAL

## References

[1] Liu J Ning X Sun X . Endoscopic sequestrectomy for skull base osteoradionecrosis in nasopharyngeal carcinoma patients: a 10‑year experience. Int J Clin Oncol 2019;24:248–255 30413913 10.1007/s10147-018-1354-8

[2] Wang L Miao J Huang H . Long-term survivals, toxicities and the role of chemotherapy in early-stage nasopharyngeal carcinoma patients treated with intensity-modulated radiation therapy: a retrospective study with 15-year follow-up. Cancer Res Treat 2022;54:118–129 34098625 10.4143/crt.2021.101 PMC8756137

[3] Miao J Di M Chen B . A prospective 10-year observational study of reduction of radiation therapy clinical target volume and dose in early-stage nasopharyngeal carcinoma. Int J Radiat Oncol Biol Phys 2020;107:672–682 32272183 10.1016/j.ijrobp.2020.03.029

[4] Lee S Choi KD Han M . Long-term outcomes of endoscopic submucosal dissection versus surgery in early gastric cancer meeting expanded indication including undifferentiated-type tumors: a criteria-based analysis. Gastric Cancer 2018;21:490–499 29052052 10.1007/s10120-017-0772-z

[5] Homma A Nakamaru Y Lund VJ . Endonasal endoscopic surgery for sinonasal squamous cell carcinoma from an oncological perspective. Auris Nasus Larynx 2021;48:41–49 33280972 10.1016/j.anl.2020.11.018

[6] Hrelec C . Management of laryngeal dysplasia and early invasive cancer. Curr Treat Options Oncol 2021;22:90 34424405 10.1007/s11864-021-00881-w

[7] Liu YP Wen YH Tang J . Endoscopic surgery compared with intensity-modulated radiotherapy in resectable locally recurrent nasopharyngeal carcinoma: a multicentre, open-label, randomised, controlled, phase 3 trial. Lancet Oncol 2021;22:381–390 33600761 10.1016/S1470-2045(20)30673-2

[8] Lee AWM Ng WT Chan JYW . Management of locally recurrent nasopharyngeal carcinoma. Cancer Treat Rev 2019;79:101890 31470314 10.1016/j.ctrv.2019.101890

[9] Li G Wang J Tang H . Comparing endoscopic surgeries with open surgeries in terms of effectiveness and safety in salvaging residual or recurrent nasopharyngeal cancer: systematic review and meta-analysis. Head Neck 2020;42:3415–3426 33463833 10.1002/hed.26397

[10] Huang Y Qiu QH . Endoscopic surgery for early-stage nasopharyngeal carcinoma: a justified initial option. Acta Otolaryngol 2017;137:1194–1198 28743209 10.1080/00016489.2017.1351041

[11] Chen MY Wen WP Guo X . Endoscopic nasopharyngectomy for locally recurrent nasopharyngeal carcinoma. Laryngoscope 2009;119:516–522 19235750 10.1002/lary.20133

[12] Liu YP Xie YL Zou X . Techniques of endoscopic nasopharyngectomy for localized stage I nasopharyngeal carcinoma. Head Neck 2020;42:807–812 31976616 10.1002/hed.26090

[13] Chen MY Wang SL Zhu YL . Use of a posterior pedicle nasal septum and floor mucoperiosteum flap to resurface the nasopharynx after endoscopic nasopharyngectomy for recurrent nasopharyngeal carcinoma. Head Neck 2012;34:1383–1388 22143978 10.1002/hed.21928

[14] You R Zou X Hua YJ . Salvage endoscopic nasopharyngectomy is superior to intensity-modulated radiation therapy for local recurrence of selected T1-T3 nasopharyngeal carcinoma – a case-matched comparison. Radiother Oncol 2015;115:399–406 25987536 10.1016/j.radonc.2015.04.024

[15] Liu YP Lv X Zou X . Minimally invasive surgery alone compared with intensity-modulated radiotherapy for primary stage I nasopharyngeal carcinoma. Cancer Commun (Lond) 2019;39:75 31730020 10.1186/s40880-019-0415-3 PMC6858734

[16] Zhang B Li Y Weng J . Efficacy and safety of endoscopic nasopharyngectomy combined with low-dose radiotherapy for primary T1-2 nasopharyngeal carcinoma. Technol Cancer Res Treat 2021;20:15330338211011975 33896244 10.1177/15330338211011975 PMC8085368

[17] Weng JJ Wei JZ Li M . Effects of surgery combined with chemoradiotherapy on short- and long-term outcomes of early-stage nasopharyngeal carcinoma. Cancer Manag Res 2020;12:7813–7826 32922081 10.2147/CMAR.S262567 PMC7457865

[18] Zalzal HG Turner MT . Robotic-assisted transmaxillary approach for removal of juvenile nasopharyngeal angiofibroma of the pterygopalatine and infratemporal fossa. Head Neck 2020;42:2745–2749 32364647 10.1002/hed.26236

[19] Ding X Lin QG Zou X . Transoral robotic retropharyngeal lymph node dissection in nasopharyngeal carcinoma with retropharyngeal lymph node recurrence. Laryngoscope 2021;131:E1895–E1902 33378575 10.1002/lary.29319

[20] Henry LE Haugen TW Rassekh CH . A novel transpalatal-transoral robotic surgery approach to clival chordomas extending into the nasopharynx. Head Neck 2019;41:E133–E140 30969009 10.1002/hed.25747

[21] Ozer E Waltonen J . Transoral robotic nasopharyngectomy: a novel approach for nasopharyngeal lesions. Laryngoscope 2008;118:1613–1616 18596562 10.1097/MLG.0b013e3181792490

[22] Tsang RK To VS Ho AC . Early results of robotic assisted nasopharyngectomy for recurrent nasopharyngeal carcinoma. Head Neck 2015;37:788–793 24604758 10.1002/hed.23672

[23] Huang L Chua MLK . Surgery as an alternative to radiotherapy in early-stage nasopharyngeal carcinoma: innovation at the expense of uncertainty. Cancer Commun (Lond) 2020;40:119–121 32189471 10.1002/cac2.12015 PMC7163772

[24] Tan R Phua SKA Soong YL . Clinical utility of Epstein-Barr virus DNA and other liquid biopsy markers in nasopharyngeal carcinoma. Cancer Commun (Lond) 2020;40:564–585 32989921 10.1002/cac2.12100 PMC7668470

[25] King AD . MR imaging of nasopharyngeal carcinoma. Magn Reson Imaging Clin N Am 2022;30:19–33 34802578 10.1016/j.mric.2021.06.015

[26] Chen ZH Lin L Wu CF . Artificial intelligence for assisting cancer diagnosis and treatment in the era of precision medicine. Cancer Commun (Lond) 2021;41:1100–1115 34613667 10.1002/cac2.12215 PMC8626610

[27] Chan KCA Woo JKS King A . Analysis of plasma Epstein-Barr virus DNA to screen for nasopharyngeal cancer. N Engl J Med 2017;377:513–522 28792880 10.1056/NEJMoa1701717

[28] Yang Z Cai J Du Z . How I do it: surgical resection of a recurrent chondromyxoid fibroma by micro-endoscopic combination technique. Acta Neurochir (Wien) 2022;164:1961–1965 35312869 10.1007/s00701-022-05185-y

[29] Zhao W Zhang D Mao X . Application of artificial intelligence in radiotherapy of nasopharyngeal carcinoma with magnetic resonance imaging. J Healthc Eng 2022;2022:4132989 35154619 10.1155/2022/4132989 PMC8828321

[30] Hsieh TY Cervenka B Dedhia R . Assessment of a patient-specific, 3-dimensionally printed endoscopic sinus and skull base surgical model. JAMA Otolaryngol Head Neck Surg 2018;144:574–579 29799965 10.1001/jamaoto.2018.0473 PMC6145784

[31] Li L Yang J Chu Y . A novel augmented reality navigation system for endoscopic sinus and skull base surgery: a feasibility study. PLoS One 2016;11:e0146996 26757365 10.1371/journal.pone.0146996 PMC4710572

