# Supplementary material for: Minimally Invasive Surgery for Early-Stage Nasopharyngeal Carcinoma
Source: J Craniofac Surg. 2022 Jul 26;33(8):e834–7. doi: 10.1097/SCS.0000000000008765 (PMC9612703; doi:10.1097/SCS.0000000000008765)
Supplement: SUPPLEMENTARY MATERIAL [file scs-33-e834-s001.docx]

**Supplemental Table 1 Results of recent studies of endoscopic surgery for early-stage NPC**

| Authors | Total of cases | | Sex  F/M | Stage  T1/T2 | With radiotherapy or/and chemotherapy | Negative surgical  margins | Follow-up  (months) | Recurrence | Distant metastasis | Survival results | Dry mouth/ sticky saliva^a^ |
| --- | --- | --- | --- | --- | --- | --- | --- | --- | --- | --- | --- |
| Huang et al, 2017 | 10 | 4/6 | | 8/2 | Chemotherapy | 100%  (10/10) | 30 | 20.0% (2/10) | None | 2-year  OS 100% | No mention |
| Liu et  al, 2019 | 10 | 3/7 | | 10/0 | None | 100%  (10/10) | 59 | None | None | 5-year  OS 100%  DMFS 100%  LRFS 100%  RRFS 100% | 3.3±10.5/  3.3±10.5 |
| Zhang et al, 2021 | 37 | 12/25 | | 15/22 | Low-dose radiotherapy | No mention | 54 | None | 2.7%  (1/37) | 5-year  OS 97.3%  DMFS 97.3%  LRFS 100%  RRFS 100% | 20.1±13.9/  15.8±11.7 |
| Weng et al, 2020 | 58 | 11/47 | | 34/24 | Radiotherapy or chemoradiotherapy | No mention | 63 | None | None | 5-year  OS 98.3%  MDFS 98.3%  DFS 98.3%  RFS 100% | No mention |

**Abbreviations:** NPC, nasopharyngeal carcinoma; F, female; M, male; OS, overall survival; DMFS, distant metastasis-free survival; LRFS, local relapse-free survival; RRFS, regional relapse-free survival; DFS, disease-free survival; RFS, relapse-free survival.

**^a^:** score assessment regarding items in QLQ-H&N35.
